# Editorial: Emotion regulation in neurodevelopmental disorders: current understanding and treatments

**DOI:** 10.3389/fpsyt.2024.1467834

**Published:** 2024-07-31

**Authors:** Ru Ying Cai, Andrea C. Samson, Mirko Uljarevic

**Affiliations:** ^1^ Aspect Research Centre for Autism Practice, Autism Spectrum Australia, Melbourne, VIC, Australia; ^2^ Olga Tennison Autism Research Centre, La Trobe University, Melbourne, VIC, Australia; ^3^ Faculty of Psychology, UniDistance Suisse, Brig, Switzerland; ^4^ Department of Special Education, Université de Fribourg, Fribourg, Switzerland; ^5^ Psychiatry and Behavioral Sciences, Stanford University, Stanford, CA, Switzerland; ^6^ Melbourne School of Psychological Sciences, The University of Melbourne, Parkville, VIC, Australia

**Keywords:** emotion regulation, neurodevelopmental disorders, intervention, mechanism, mental health

Emotion regulation is a transdiagnostic process that plays a pivotal role in the development and maintenance of internalizing and externalizing symptoms such as anxiety, depression, or conduct problems. Difficulty with emotion regulation is a prominent feature of neurodevelopmental conditions such as autism spectrum disorder (ASD) and attention deficit hyperactivity disorder (ADHD), as well as neurogenetic syndromes. This Research Topic, *Emotion Regulation in Neurodevelopmental Disorders: Current Understanding and Treatments*, aimed to bring together theoretical insights, methodological developments, and novel empirical findings of emotion regulation in individuals diagnosed with neurodevelopmental disorders. In total, nine articles were published. These papers covered a range of important topics that could be broadly organized into studies focused on the role and mechanisms underlying emotion regulation difficulties and trials evaluating the effectiveness of different intervention modalities on emotion regulation-related outcomes. It was particularly encouraging that one study focused on measurement development and that several studies (both mechanistic and intervention-focused) were conducted with individuals with neurogenetic syndromes. Below, we provide a brief overview of the main findings of the published studies.

## Studies focused on interventions related to emotion regulation

A single-subject design study involving four children with Williams Syndromes enrolled in play- and humor-infused exposure therapy approach showed preliminary evidence for improvements in emotion regulation (Young et al.). More specifically, at the conclusion of the brief intervention, children with Williams Syndrome who presented with baseline co-occurring fears and phobias showed improved tolerance or the ability to engage positively with the previously real-world feared stimulus. A study by Famelart et al. evaluated EMO-T, an intervention aimed at improving emotion expression, recognition, comprehension, and regulation, hypothesized as skills essential for the healthy regulation of emotions that, if impaired, could result in dysregulation difficulties commonly observed in children with Prader–Willi Syndrome (PWS). In addition to improvements in voluntary expression and emotion recognition abilities, school-aged children with PWS enrolled in EMO-T also showed improvements in emotion regulation based on the parent-reported Emotion Regulation Checklist. Enav et al. evaluated whether participation in a four-week reflective/mentalization-based parenting workshop can enhance emotion regulation in parents of children with ASD. Authors specifically focused on different aspects of cognitive reappraisal given its adaptive role in reducing levels of stress, a particularly pertinent issue for parents of children with autism. Relative to the baseline, parents reported higher levels of cognitive reappraisal, as indexed by the Emotion Regulation Questionnaire, as well as higher levels of reflective (but not non-reflective) reappraisal on the Emotion Interaction Questionnaire. Finally, in a large sample of 94 children with attention-deficit/hyperactivity disorder (ADHD), Groves et al. reported that two digital training approaches focused on working memory (Central Executive Training) and inhibitory control (Inhibitory Control Training) both resulted in a significant reduction in emotion dysregulation (measured by the Emotion Dysregulation Inventory) at immediate post-treatment, at 1–2 month and 2–4-month follow-ups.

## Studies focused on characterization and the role of emotion regulation

In the only study specifically focused on measurement development, Uljarević et al. described the development, refinement, and initial psychometric evaluation of a new open-source 52-item Executive Functioning Scale (EFS) that was specifically developed to capture theoretically-based facets of executive functioning (EF), including working memory and sequencing, response inhibition, set-shifting, processing speed, risk avoidance, and crucially, emotion regulation. Across two independent data collections encompassing 2,958 children and adolescents, the hypothesized six subscales were derived and confirmed, showing strong classic test and conditional reliability as well as invariance across age, sex, race, and ethnicity groups. Walter et al. explored the association between affective language, as measured by the verbal fluency test, with emotion dysregulation, indexed by the cyclothymic dimension of the Temperament Evaluation of Memphis questionnaire, in adults with ADHD, ASD with co-occurring ADHD, and neurotypical controls. Findings provided very preliminary suggestions that factors related to emotional difficulties in individuals with ADHD and with ASD with co-occurring ADHD might be at least somewhat distinct. More specifically, compared to adults with ADHD and neurotypical controls, individuals with ASD and ADHD produced fewer anger-related words, produced more emotions, and fewer rule breaks. Authors reported associations with emotion regulation only in adults with ADHD, finding that emotional over-reactivity in adults with ADHD was associated with the number of emotions and the frequency of these words. Comparing the effects of the COVID-19 pandemic between 4,138 children with ASD and 711 children with developmental delays (DD), Zhao et al. found that children with ASD had a higher risk of having more emotional and behavioral problems than children with DD. De Blasio et al. explored olfactory processing in 20 youth with profound intellectual and multiple disabilities, demonstrating preserved olfactory preferences in this population and, importantly, that olfactory preferences were associated with mood levels. Samson et al. aimed to characterize emotion regulation strategy use and its associations with anxiety use in individuals with ASD with (*n*=785) and without ID (*n*=596), Williams Syndrome (*n*=261), and Intellectual Disability not otherwise specified (ID-NOS; *n*=649). Distinct emotion regulation use was identified such that individuals with Williams Syndrome most frequently used parent routine, parent shielding, repetitive behaviors, and distraction; individuals with ASD without ID most frequently utilized isolation/withdrawal; and in general, individuals with ASD and ID and with ID-NOS engaged significantly less frequently in cognitive emotion regulation strategies compared to other groups. Importantly, across all groups, anxiety was linked to a higher use of maladaptive and a lower use of positive strategies.

## Conclusion

The research studies included in this Research Topic provided important insights into the transdiagnostic nature of emotion regulation across various neurodevelopmental conditions, providing important data that can be used across both research and clinical contexts. Crucially, several key directions for future research have emerged, including the need for a more careful conceptualization of emotion regulation and the development of new multimodal assessment protocols. Additionally, improving the design of interventions and utilizing adaptive trial frameworks are essential steps toward precision medicine for improving the mental health of people with neurodevelopmental conditions.

## Author contributions

RC: Writing – review & editing. AS: Writing – review & editing. MU: Writing – original draft.

